# Unveiling the cognitive network organization through cognitive performance

**DOI:** 10.1038/s41598-024-62234-5

**Published:** 2024-05-21

**Authors:** A. Borne, C. Lemaitre, C. Bulteau, M. Baciu, M. Perrone-Bertolotti

**Affiliations:** 1 Univ. Grenoble Alpes, Univ. Savoie Mont Blanc, CNRS, LPNC, 38000 Grenoble, France; 2 Service de Neurochirurgie Pédiatrique, Hôpital Fondation Adolphe de Rothschild, 75019 Paris, France; 3 MC2 Lab, Institut de Psychologie, Université de Paris-Cité, 92100 Boulogne-Billancourt, France

**Keywords:** Language, Psychology

## Abstract

The evaluation of cognitive functions interactions has become increasingly implemented in the cognition exploration. In the present study, we propose to examine the organization of the cognitive network in healthy participants through the analysis of behavioral performances in several cognitive domains. Specifically, we aim to explore cognitive interactions profiles, in terms of cognitive network, and as a function of participants’ handedness. To this end, we proposed several behavioral tasks evaluating language, memory, executive functions, and social cognition performances in 175 young healthy right-handed and left-handed participants and we analyzed cognitive scores, from a network perspective, using graph theory. Our results highlight the existence of intricate interactions between cognitive functions both within and beyond the same cognitive domain. Language functions are interrelated with executive functions and memory in healthy cognitive functioning and assume a central role in the cognitive network. Interestingly, for similar high performance, our findings unveiled differential organizations within the cognitive network between right-handed and left-handed participants, with variations observed both at a global and nodal level. This original integrative network approach to the study of cognition provides new insights into cognitive interactions and modulations. It allows a more global understanding and consideration of cognitive functioning, from which complex behaviors emerge.

## Introduction

Cognition encompasses a multitude of intricate functions and processes that enable the human thinking, perception, and behavior within the world. These functions include language, memory, executive functions, and social cognition among others. Although they have been studied in isolation over decades, these cognitive functions are inherently interwoven, a phenomenon demonstrated throughout life. Typically, during development, cognitive functions progressively mature and specialize within distinct brain networks^1–3^. This developmental trajectory emphasizes the interactive nature of cognitive maturation, where emerging functions rely on those already developed. For instance, relationships between the development of executive functions and language have been demonstrated in early childhood^4–7^, suggesting that interactive phenomena lead to the emergence of mature cognition. This phenomenon of cognitive interaction is dynamic across the lifespan. Indeed, in adulthood, performing a task and reasoning on a fact inherently involve the orchestration of multiple cognitive functions through large-scale networks, precluding the strict isolation of one process from others when analyzing behaviors^8–16^.

Consequently, a more integrative approach becomes necessary in order to investigate human cognitive functioning. This paradigm shift has already occurred in the field of neuroimaging, transitioning from a localizationist stance to a connectomic perspective^17^. Brain regions are no longer considered isolated entities, but rather nodes within a dynamic and complex network^18–20^. Similarly, an integrative and network perspective is emerging within cognitive research. Cognitive functions are now acknowledged as interwoven systems, operating as a network^21^, deeply intertwined within the brain's structural and functional connectomes. Recently, Roger et al. proposed the term “cognitome”, a holistic perspective that takes into account the associations between brain connectivity and cognitive efficiency when considering human neurocognitive functioning in an integrative way^22^. A significant overlap and flexible integration of different brain networks has been demonstrated during cognitive tasks of different levels of complexity, providing further evidence of the integrative functioning of large-scale networks in human cognition^23^. In this theoretical framework, cognition must be viewed from a broader perspective, akin to a network, and the term 'cognitome' can generally encompass the understanding of cognition as a vast interactive system.

In this context, examining cognition as a network is of particular interest^24^. For instance, by conducting network analyses, a mutualism model of intelligence has been postulated^25^. This approach effectively models the dynamic mutual interactions among cognitive functions that underlie intelligence. This network-based perspective provides evidence for a mutualism concept of cognition, asserting that cognitive functions interact, and the development of one function depends on its own progress as well as that of the other functions^26^. In this framework, and using methods like graph theory, cognitive functions can be viewed as nodes within an extensive network, interconnected by edges that delineate the network's architecture and model cognitive interactions^27^. The network approach is thus well-suited for uncovering subtleties in cognitive functioning, moving beyond the description of performances and delving into cognitive organization and interactions across domains^28^. Indeed, this more systemic and integrative approach to cognition enables a more comprehensive evaluation of its dynamics and organization. Through network methodologies approaches such as graph theory, studies have been able to elucidate specific cognitive network (dis)organization in the presence of a pathology^29–31^, as well as across the lifespan^32,33^. These findings underscore the importance of extending beyond behavioral performances to gain a deeper understanding of cognitive functioning and the underlying systems interactions. In this context, it appears crucial to adopt this perspective in the study of cognition, even within healthy functioning. Thus, we can expect to uncover diverse cognitive profiles, as supported by different systems interactions or different networks structure that may elude detection through isolated cognitive score assessments alone. Understanding how diverse factors influence interactions within the network can offer valuable insights into the understanding of cognition and the emergence of complex behaviors.

In this regard, a characteristic of specific interest is manual preference or handedness, as defined by the hand preferentially used to write or perform various manual actions in daily life, where differences in terms of brain representation and cognitive performance remain controversial. Around 90% of the population presents right hand lateralization^34^. Traditionally, handedness has been considered an indicator of cerebral organization and, in particular, language lateralization^35^. However, more recent neuroimaging studies have qualified these findings. Indeed, both right-handed and left-handed participants typically exhibit left hemisphere specialization for language^36,37^. Nevertheless, there is a higher prevalence of left-handers in people presenting atypical hemispheric asymmetries^36,38^. For instance, in individuals with atypical language organization, that is bilateral or right specialization for language, left-handers are much more widely represented than in individuals presenting typical organization^39^. However, while some differences may have been demonstrated at the cerebral level in specific cases, the impact of handedness on cognitive performance remains elusive, if not negligible^40,41^. By employing a more integrative approach in the study of cognition, and considering the cognitive system as a whole, we could compare its organization and highlight variability in functioning that may not necessarily be reflected by performance alone. To our knowledge, no study has investigated the difference in cognitive profiles between left-handed and right-handed participants by adopting a network perspective. This approach could enable a more in-depth study of the extent to which this variable can modulate cognitive organization at a more global level.

The aim of this study is therefore to investigate cognitive organization from an integrative perspective, employing graph theory methodologies. Specifically, using this network approach, we want to reveal the interactions between language, memory, executive functions, and social cognition manifested at a behavioral level. By surpassing mere behavioral assessments, we aim to evaluate whether handedness is associated with the organization of the cognitive network. The main goal of the current study is to go beyond behavioral performance in the study of cognition and to use novel approaches to move towards a cognitomic perspective. This model of understanding emphasizes the pertinence of a network-based approach among healthy subjects, thereby deepening our comprehension of cognition and its interactions.

## Method

### Participants

One hundred and seventy-five young healthy adults (Mean age = 20.57y; SD = 2.08y) participated in the study. They were native French speakers and exhibited normal or corrected-to-normal vision. Participants included 100 women (50 right-handed and 50 left-handed) and 75 men (50 right-handed and 25 left-handed). Handedness was evaluated using the Edinburgh Handedness Inventory^42^. To perform a comparative analysis of performance and cognitive network organization based on handedness, 75 of the 100 right-handed participants were randomly selected (Mean age = 20.6y; SD = 2.07y) and paired in terms of gender and age with the 75 left-handed participants included in the sample (Mean age = 20.59y; SD = 2.13y). Most of the participants were graduate and undergraduate students from Grenoble Alpes University and received course credit at the university for their participation. This research has been performed in accordance with the Declaration of Helsinki. All methods were performed in accordance with relevant guidelines and regulations and participants provided informed consent to take part in the study. The research was approved by the Rothschild Foundation Hospital review board – IRB 00,012,801- under the study number CE_20201124_1_CBU.

### Cognitive evaluation

The cognitive functions assessment was conducted using the computerized behavioral task battery LEXTOMM^43^. This battery included multiple tasks developed with E-prime 3, allowing language, executive functions, theory of mind, and memory evaluation. Specifically, 10 cognitive functions were assessed in this study. The language assessment comprised four different tasks assessing semantics, phonological, syntactic, and prosodic abilities. These tasks were respectively a categorization task in which participants were instructed to judge whether verbal items represented living or non-living entities, a rhyme detection task where participants judged whether two pictures corresponded to words that rhymed, a sentence-picture matching task requiring to ascertain if the presented picture matched the preceding heard sentence, which could be active or passive and affirmative or negative, and a focus detection task requiring to judge whether auditory sentences contained a contrastive focus. Executive functions were evaluated through four tasks. Inhibition was assessed via a Flanker task, where participants determined whether a target arrow amidst distracting arrows, pointed right or left. The working memory task was an N-back task requiring participants to determine if a letter matched the second-to-last one shown. Switching abilities were assessed through a task involving alternating between categorizing letters (vowel vs. consonant) and numbers (even vs. odd). Sustained attention was then assessed using a sustained attention to response task (SART), which demanded Go/No-Go responses on numeric stimuli. Finally, memory and Theory of Mind (ToM) abilities were respectively assessed through a verbal declarative memory task where participants indicated if an auditory word was previously shown in non-verbal modality in a preceding task, and a false beliefs attribution task requiring participants to select the correct ending of a short video in a forced-choice paradigm. A comprehensive description of the experimental protocol is available at https://osf.io/hkwdb/. Participants were tested individually in a quiet room and sat in front of a computer screen, at 50 cm from the display. They indicated their behavioral response manually on a computer mouse with their dominant hand. Behavioral performances, including response accuracy (% of correct responses) and reaction times (in ms) were recorded across all tasks.

### Data analysis

#### Cognitive performances

For each participant and each cognitive task, accuracy and reaction times (RT) were collected and subsequently transformed into z-scores. Prior to analysis, scores with an accuracy below 50% were omitted from the analysis, as they indicate that the task was not executed accurately and responses were provided randomly. In order to perform a combination of both accuracy and reaction time, we compute the RT z-score as the opposite of the mean reaction times, so that a higher score always corresponded to a higher performance. To capture with a larger precision the participants’ performance, we then computed a general composite z-score for each participant, reflecting the mean z-score across accuracy and reaction times. This average z-score was included as the dependent variable in data analysis.

To evaluate whether handedness is associated with cognitive performance, we computed a Wilcoxon two-sample test, comparing the right-handed and left-handed participants' performance scores (accuracy, RT, and mean z-score) across all the cognitive tasks. *P*-values were adjusted with a Bonferroni correction for multiple comparisons (Table 1).

**Table 1:** Distribution of scores for right- and left-handed participants for each task and each measure.

	Task	Score	Right-handed	Left-handed	Wilcoxon test
		Mean (SD)	(n = 75)	(n = 75)	
Language	Semantics	%CR	95.88 (4.63)	95.58 (6.84)	*p* = .67
		RT (ms)	662.94 (100.84)	679.51 (124.24)	*p* = .61
		Mean Z	0.05 (0.45)	-0.05 (0.74)	*p* = .85
	Syntax	%CR	89.06 (5.38)	85.67 (9.85)	*p* = .08
		RT (ms)	1255.18 (181.18)	1319.84 (230.78)	*p* = .06
		Mean Z	0.22 (0.60)	-0.14 (0.98)	*p* = .06
	Phonology	%CR	89.02 (10.39)	89.38 (8.68)	*p* = .76
		RT (ms)	1567.29 (262.39)	1573.44 (243.92)	*p* = .96
		Mean Z	0.06 (0.92)	0.07 (0.81)	*p* = .76
	Prosody	%CR	90.24 (6.66)	89.37 (9.03)	*p* = .88
		RT (ms)	346.65 (101.29)	367.58 (127.67)	*p* = .57
		Mean Z	0.14 (0.51)	0.01 (0.72)	*p* = .61
	Memory	%CR	91.89 (4.66)	89.50 (8.04)	*p* = .17
		RT (ms)	1647.68 (128.90)	1627.65 (120.86)	*p* = .27
		Mean Z	0.07 (0.65)	-0.03 (0.84)	*p* = .99
Executive functions	Inhibition	%CR	96.76 (3.38)	95.51 (4.37)	*p* = .06
		RT (ms)	546.71 (90.83)	520.83 (66.40)	*p* = .13
		Mean Z	0.01 (0.54)	-0.01 (0.70)	*p* = .67
	Working memory	%CR	87.24 (7.12)	85.78 (7.24)	*p* = .45
		RT (ms)	702.46 (244.41)	738.49 (224.73)	*p* = .20
		Mean Z	0.12 (0.80)	-0.07 (0.74)	*p* = .45
	Switching	%CR	94.79 (7.15)	94.98 (7.64)	*p* = .56
		RT (ms)	1300.37 (278.61)	1393.71 (350.22)	*p* = .10
		Mean Z	0.10 (0.67)	-0.03 (0.78)	*p* = .42
	Attention	%CR	91.69 (8.21)	89.12 (9.33)	*p* = .08
		RT (ms)	427.51 (77.78)	408.89 (90.93)	*p* = .26
		Mean Z	0.01 (0.53)	-0.02 (0.61)	*p* = .99
	Theory of Mind	%CR	92.96 (6.13)	93.03 (9.81)	*p* = .21
		RT (ms)	2347.90 (920.91)	2311.20 (796.15)	*p* = .85
		Mean Z	0.04 (0.68)	0.07 (0.72)	*p* = .63

#### Inter-cognitive evaluation: graph analyses

In this study, we were specifically interested in investigating the intricate structure of the interplay between different cognitive functions and their mutual influences within the cognitive network. To this aim, we first computed the correlations between the behavioral performances across all tasks. Specifically, we performed Spearman correlations on the mean of the composite z-scores, previously computed This generated a correlation matrix that was used for subsequent network analyses. P-values were adjusted with the Holm correction for multiple comparisons. Using this approach, we generated for the entire sample (n = 175), the sub-group of right-handed participants (n = 75) and the sub-group of left-handed participants (n = 75) a correlation matrix that was used for subsequent network analyses.

**Figure 1:**
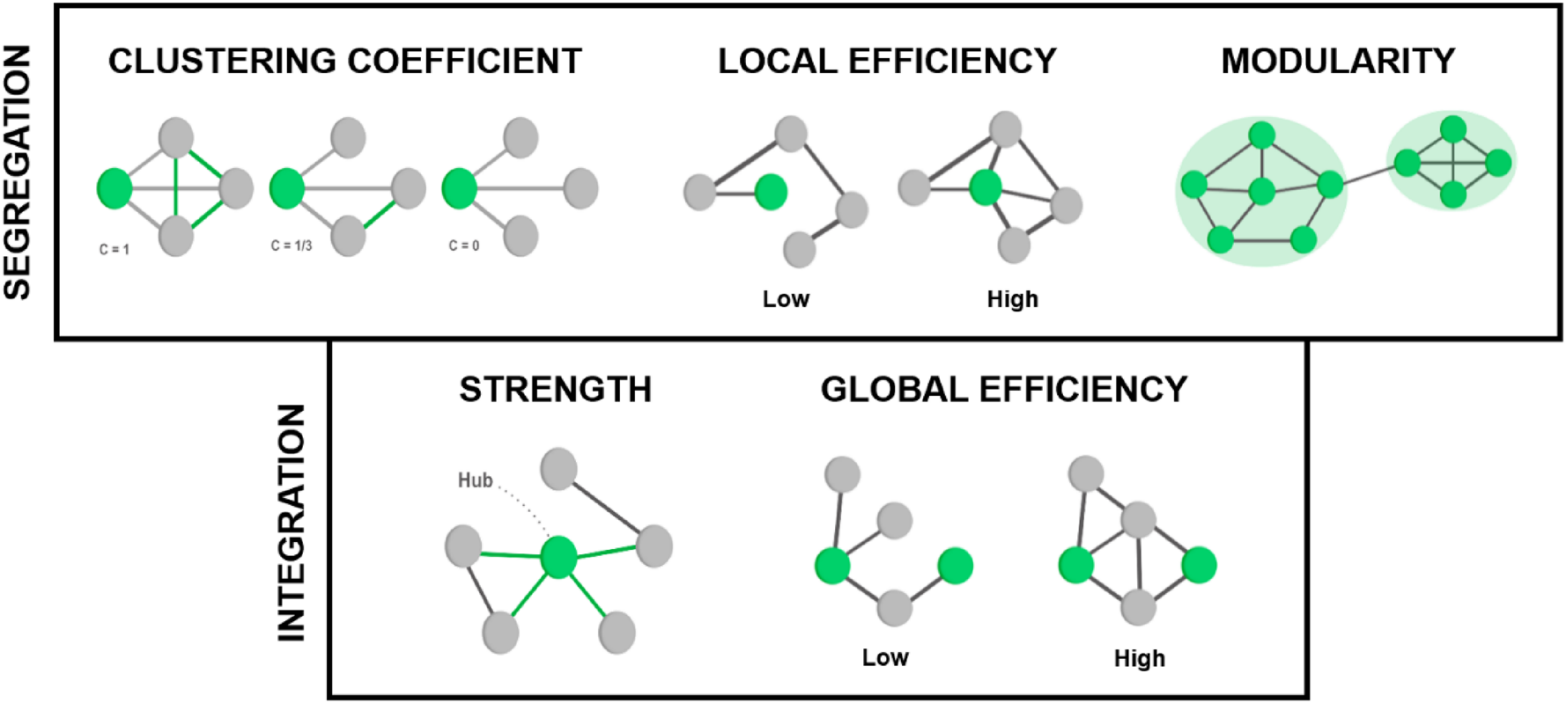
Schematic representations of graph theory metrics used to describe the organization of a network. *Note:* Segregation Metrics: The clustering coefficient reflects the tendency of two neighbors of a given node to be connected, forming a triangular relationship. Local efficiency, on the other hand, measures a node's ability to transmit information effectively to its direct neighbors. It is calculated as the average of the inverse of the shortest paths between the neighbors of a node and the rest of the network. Modularity indicates the tendency of a network to organize into different communities, where nodes within the same community are more strongly connected than nodes in different communities. Integration Metrics: Node strength is defined as the sum of the weights of the edges connecting it to the rest of the network. Global efficiency assesses a network's ability to transmit information effectively among all its nodes, calculated as the average of the inverse distances between all pairs of nodes within the network.

Graph analyses were carried out using the NetworkToolbox package^44^ available in RStudio and the GraphVar toolbox^45^ in MATLAB. The cognitive network explored here encompassed ten nodes, corresponding to the ten assessed cognitive tasks, with edges representing the absolute value of correlation coefficients linking the respective cognitive tasks. To reduce the risk of spurious associations and maintain consistency in the number of nodes and edges across networks, facilitating comparisons, we applied the triangulated maximally filtered graph (TMFG) approach^47^ as described by Christensen and colleagues^46^. Given the study's focus on unraveling the cognitive network's architecture within healthy participants, global and local graph metrics were extracted to provide insights into network characteristics. Local metrics encompassed node-specific attributes such as strength, clustering coefficient, and local efficiency. Meanwhile, global metrics included parameters like global clustering coefficient, global efficiency, and modularity. Figure 1 provides a schematic representation of these metrics.

To assess stability measures and facilitate statistical comparisons among groups, we employed a bootstrapping approach. Specifically, we conducted case-wise bootstrapping, resampling each group's data (i.e., all participants, right-handed, and left-handed) 1000 times with replacement, resulting in 1000 matrices per group from which networks were derived using the aforementioned procedure. Therefore, for each group, we obtained the matrix and network based on the empirical data (i.e., empirical network) and 1000 matrices and networks (i.e., resampled networks) derived from the bootstrap procedure. Subsequently, graph theory metrics were computed for each network, enabling the derivation of a reference distribution of each metric for each group and facilitating statistical inference of differences between groups.

To verify the network metrics stability obtained from empirical networks within each group, we computed the 95% confidence interval (CI95) based on the resampled networks. Values observed outside the CI95 confidence interval indicated significant deviations from the resampled distribution, highlighting a lack of stability in the measurement. Finally, to statistically examine network organization differences between right-handed and left-handed individuals, we compared the distribution of both groups for each metric using *t-*tests, Bonferroni-corrected for multiple comparisons. All analyses were conducted using RStudio (R version 4.0.3), with an alpha threshold set at 0.05.

Visual representation of the cognitive network was allowed by reconstructing the graph from the correlation matrix using *Gephi* software (https://gephi.org/), adopting the Force Atlas 2 algorithm for spatialization. Node color denoted cognitive domains (i.e., language, executive functions, episodic memory, and theory of mind), size was proportional to strength, and edge thickness indicated the strength of correlation coefficients.

## Results

### Cognitive scores

**Figure 2:**
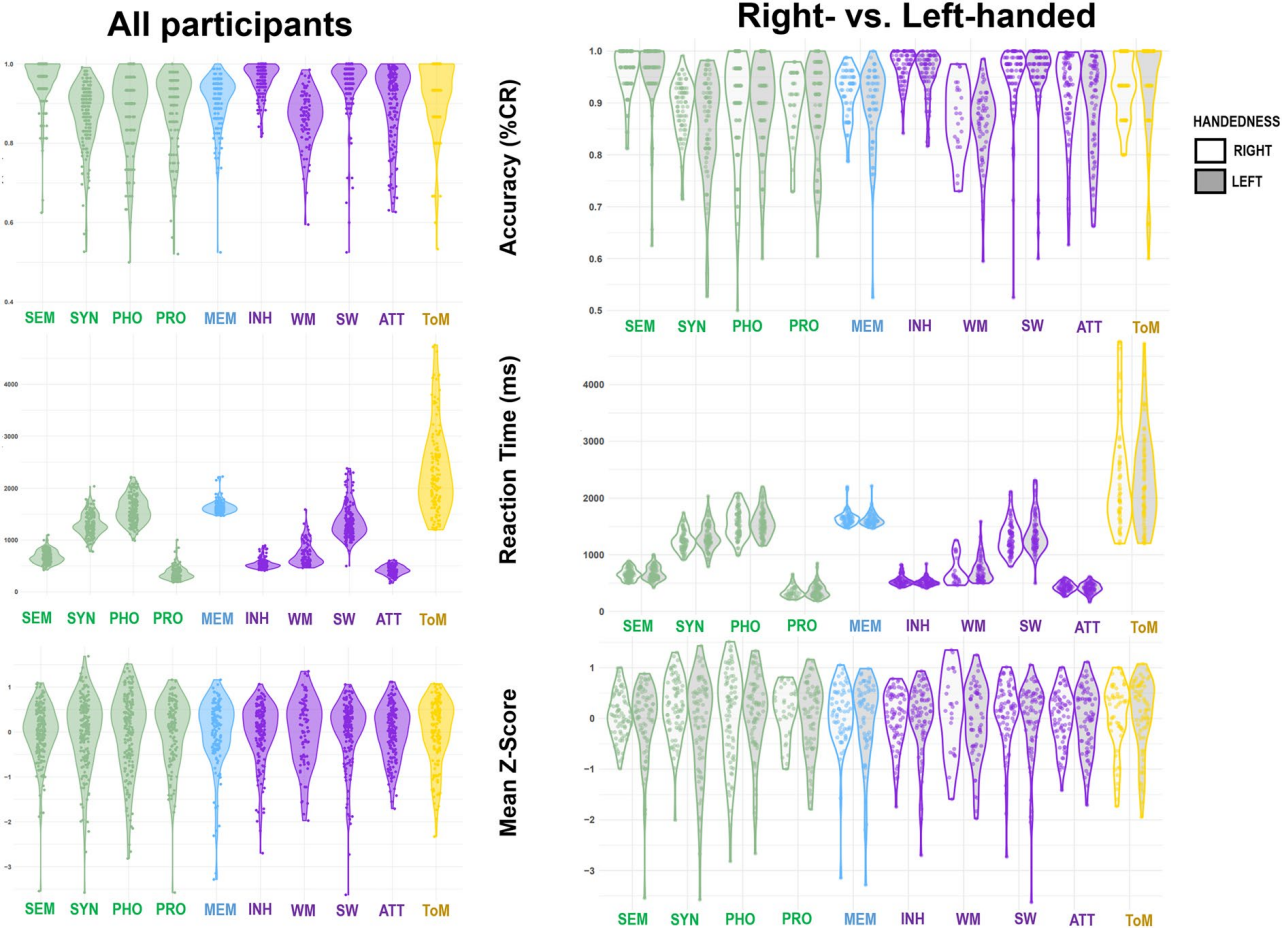
Cognitive scores on each cognitive domain for all participants and according to handedness. *Note*: Results are presented in terms of accuracy, reaction times and mean Z-Scores for all participants (on the left, n = 175) and in line with handedness (on the right, n = 150, Right = 75, Left = 75). Right-handed and left-handed participants are respectively represented by white and grey violins*. Abbreviations*: SEM: semantics; SYN: syntax; PHO: phonology; PRO: prosody; MEM: episodic memory; INH: inhibition; WM: working memory; SW: switching; ATT: sustained attention; ToM: theory of mind.

Participants exhibited high performance across all cognitive functions, suggesting that all participants performed correctly all the behavioral tasks. Mean accuracy ranged from 86.4% of correct responses (working memory) to 96.2% (inhibition), and mean reaction times spanned from 363.99 ms (prosody) to 2368.10 ms (ToM). The distributions of scores are presented in Fig. 2.

No significant differences were found between the right-handed and the left-handed participant groups across any tasks, either in terms of accuracy, RT, or mean z-score, suggesting that both groups performed similarly in all behavioral tasks. Detailed score distribution and results of the Wilcoxon tests are documented in Table 1.

### Inter-cognitive evaluation: graph analyses

**Figure 3:**
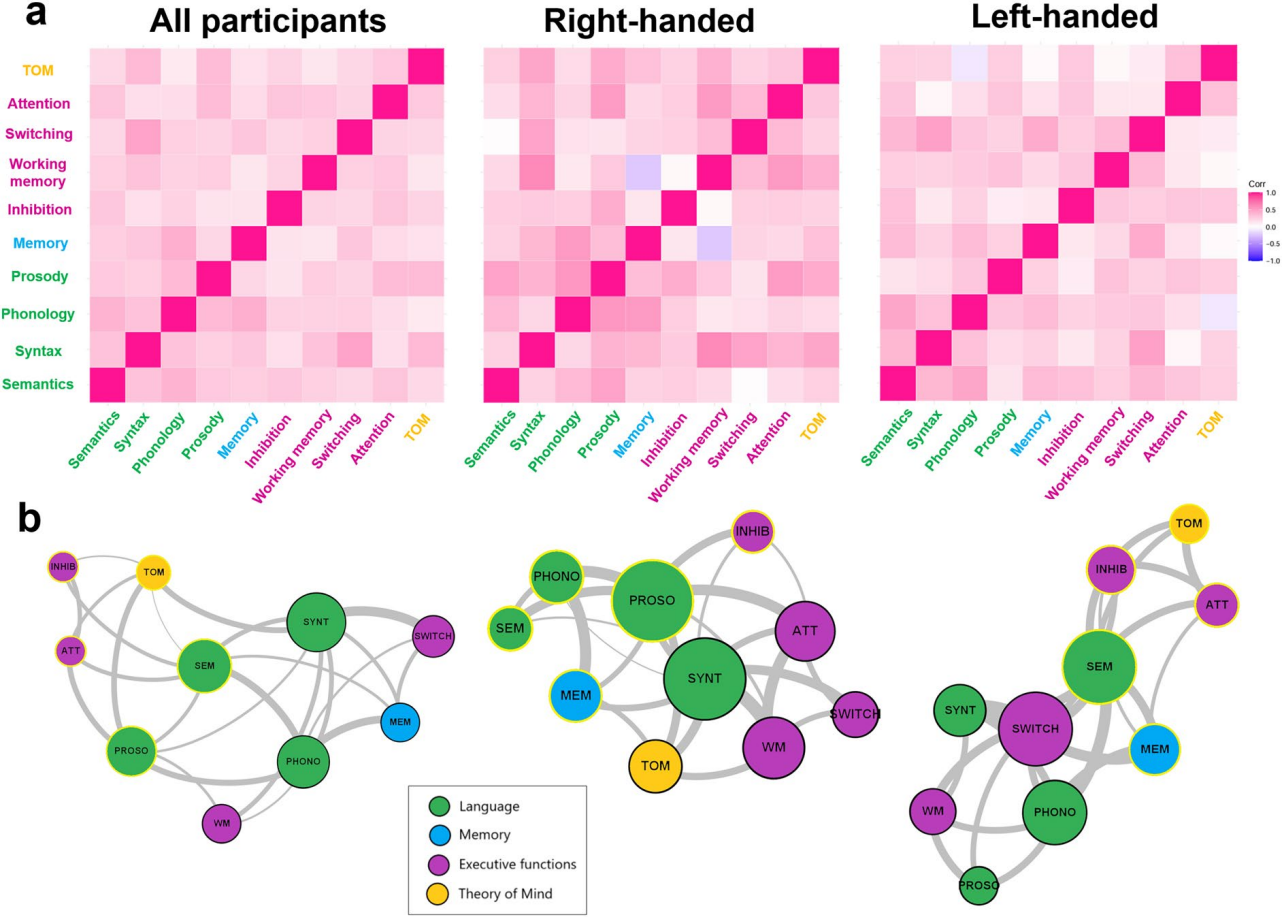
Cognitive interaction networks. *Note*: The Panel (**a**) shows the correlations between scores (mean z-scores) from LEXTOMM cognitive tasks for all participants and for each of handedness group. The Panel (**b**) shows the graph representations of the derived cognitive networks. The size of the nodes represents the strength, and the thickness of the edges is proportional to the correlation coefficient between the two scores. Communities highlighted in the network are represented by the color of the node outline (i.e., black and yellow).

Correlation analyses performed in the entire population revealed interactions between z-scores derived from the diverse cognitive tasks (Fig. 3a). Specifically, 19 under 45 significant positive correlations were found after a multiple comparisons correction, with coefficients ranging from 0.26 to 0.49. Higher correlations were found between syntax and switching (*r* = 0.49, *p* < 0.001), phonology and memory (*r* = 0.43, *p* < 0.001), semantics and phonology (*r* = 0.41, *p* < 0.001), phonology and prosody (*r* = 0.37, *p* < 0.05), and syntax and ToM (*r* = 0.37, *p* < 0.001). Within the same cognitive domain, language tasks were correlated with each other, but this was not the case for all tasks belonging to the executive domain. Nevertheless, significant correlations also occurred between cognitive functions belonging to different domains.

Different patterns of correlations were observed when the handedness of the participants was considered (Fig. 3a). After Holm corrections, 6 significant correlations were found for right-handed participants. These correlations were found between phonology and prosody (*r* = 0.55, *p* < 0.05), phonology and memory (*r* = 0.54, *p* < 0.001), syntax and switching (*r* = 0.49, *p* < 0.001), syntax and ToM (*r* = 0.48, *p* < 0.05), syntax and attention (*r* = 0.4, *p* < 0.05), and phonology and semantics (*r* = 0.37, *p* < 0.05). Regarding the left-handed participants, 5 significant correlations were observed, between syntax and switching (r = 0.50, p < 0.001), semantics and phonology (r = 0.48, p < 0.001), memory and switching (r = 0.45, p < 0.05), semantics and syntax (r = 0.39, p < 0.05), and semantics and switching (r = 0.38, p < 0.05). For both right- and left-handed participants, some correlations between functions belonging to different domains have been observed, but not all language functions correlated with each other, nor executive functions.

**Figure 4:**
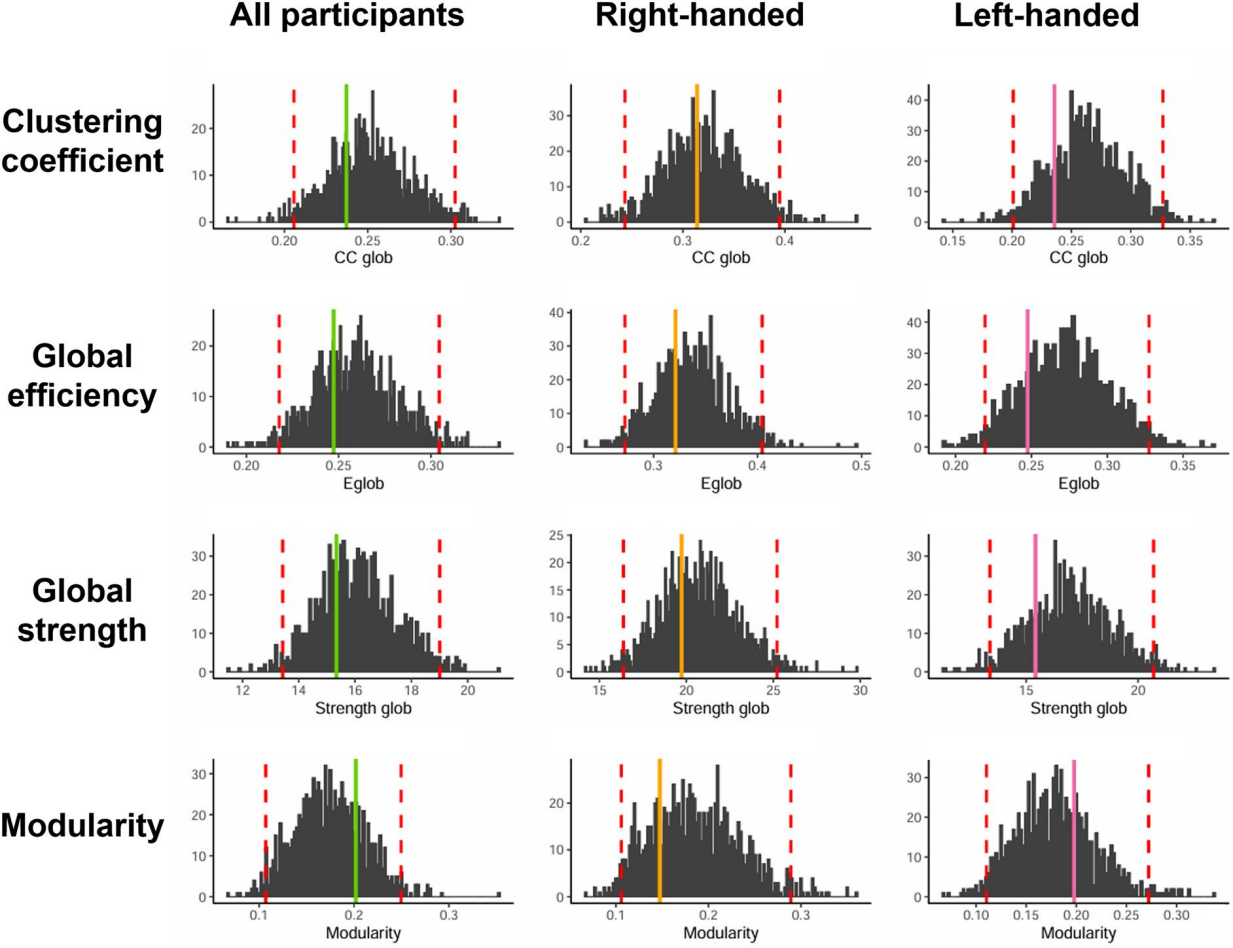
Global metrics distribution for all participants and the right-handed and left-handed groups. *Note*: Solid lines indicate the metric extracted for the empirical network of each group. Red dotted lines indicate the 95% confidence interval calculated based on the distribution of the metrics from the resampled networks.

From correlations, graphs representing the empirical cognitive network were constructed (Fig. 3b). Global metrics of empirical networks are presented in Table 2. For each group, all metrics extracted from empirical networks were inside the CI95 interval computed on resampled networks, highlighting stability in the metric estimation (Fig. 4).

**Table 2:** Global network metrics for all participants and the right-handed and left-handed groups.

	All participants		Right-handed		Left-handed	
	*Empirical*	*CI-95%*	*Empirical*	*CI-95%*	*Empirical*	*CI-95%*
Clustering coefficient	0.2371	[0.206–0.302]	0.3140	[0.243–0.395]	0.2357	[0.201–0.327]
Global efficiency	0.2473	[0.218–0.305]	0.321	[0.272–0.404]	0.2475	[0.219–0.328]
Global strength	15.3373	[13.4–19.0]	19.726	[16.4–25.2]	15.4139	[13.4–20.7]
Modularity	0.2019	[0.107–0.250]	0.1465	[0.106–0.289]	0.1978	[0.11–0.272]

The 95% confidence intervals (CI95) were computed from the 1000 resampled networks.

**Figure 5:**
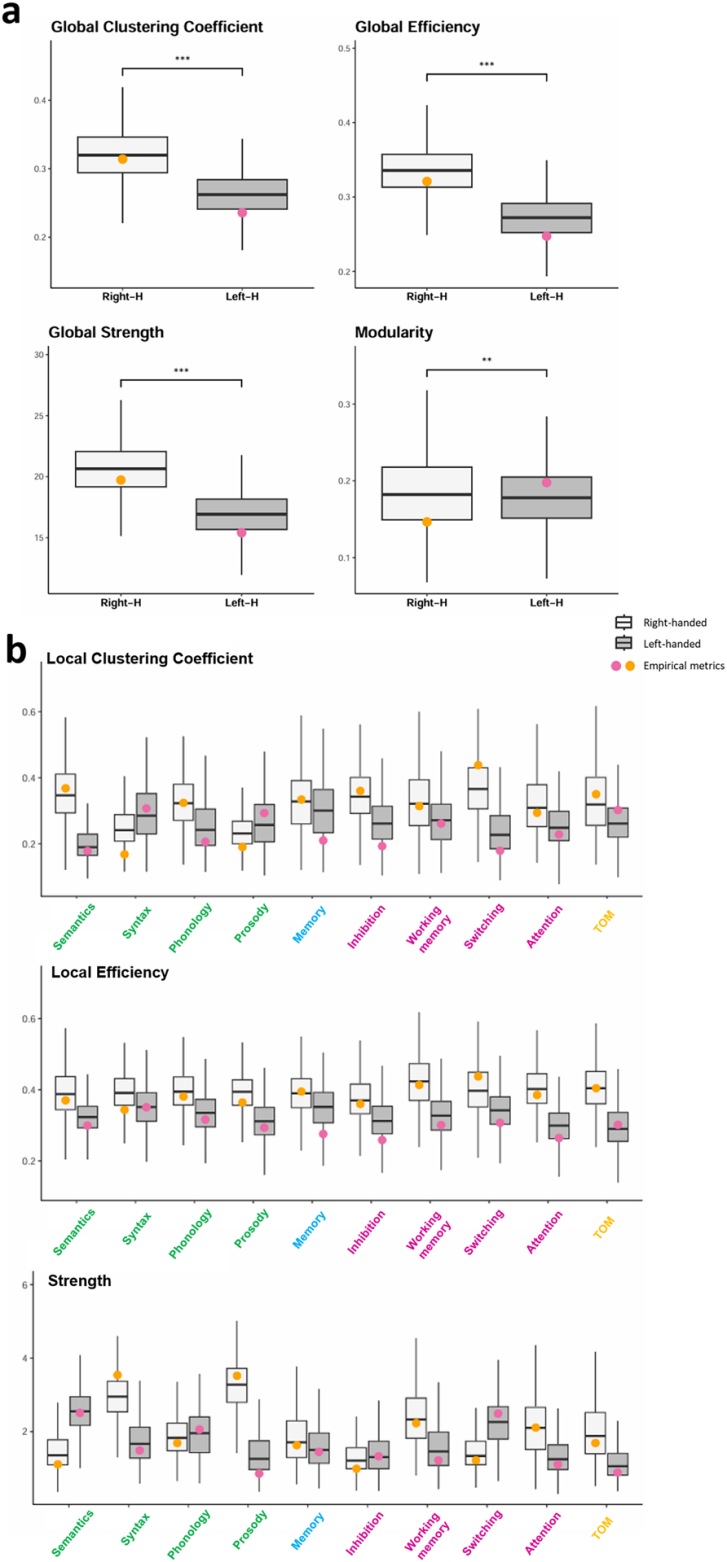
Comparison of cognitive network metrics between right-handed and left-handed participants. *Note*: The Panel (**a**) shows the Global metrics extracted for right- and left-handed participants (Right-H and Left-H, respectively). The Panel (**b**) shows the local metrics reflecting properties of segregation (clustering coefficient, local efficiency) and integration (strength) for each of the cognitive tasks assessed. Boxplots depict the distribution of each metric derived from the bootstrapping procedure. Colored points represent the metric values obtained from the empirical network of both right-handed and left-handed participants.

We applied case-wise bootstrapping procedures to generate the distribution of each metric for both groups and statistically analyzed the difference in the structure of cognitive networks of right-handed and left-handed participants. Among all participants, functions demonstrating higher integration assessed by strength included syntax, phonology, semantics, and prosody, while functions that present higher levels of segregation (assessed through local efficiency and local clustering coefficient) encompassed memory, working memory, switching, inhibition, and ToM. Furthermore, this study aimed to use this approach to analyze the difference in the cognitive networks structure of right-handed and left-handed participants. At a global level, metrics showed differences in terms of integration and segregation of the network (Fig. 5a). Both global efficiency (*t* = -44.98, *p* < 0.001) and global clustering coefficient (*t* = -36.01, *p* < 0.001) appeared to be more important in the graphs of right-handed compared to those of left-handed participants. Similarly, modularity (*t* = -3.00, *p* < 0.05) and global strength (*t* = -40.85, *p* < 0.001) were significantly higher in the networks of right-handed participants than in those of left-handed participants, underscoring disparities in the cognitive network's organization.

Graph metrics were also extracted at the local level (Fig. 5b). Functions displaying higher integration, as assessed by strength, exhibited variability based on handedness. Significantly different strengths were observed for each function assessed, except for phonology. Across bootstrapped samples, left-handed individuals demonstrated higher strength compared to right-handed individuals in semantics, inhibition, and switching. Among right-handed participants, prosody and syntax displayed the strongest integrative properties, followed by working memory. Conversely, in left-handed participants, semantics exhibited the highest strength, followed by switching and phonology.

Regarding segregation properties within the network, functions showing enhanced segregation, as indicated by a higher clustering coefficient, included switching, semantics, and inhibition for right-handed participants, and memory, syntax, and working memory for left-handed participants. Right-handed participants exhibited higher clustering coefficients compared to left-handed participants for each function, except for syntax and prosody, for which left-handed participants displayed higher coefficients. Moreover, functions demonstrating higher local efficiency comprised working memory, ToM, and attention for right-handed participants, and syntax, memory, and switching for left-handed participants. Right-handed participants demonstrated higher local efficiency compared to left-handed participants across all cognitive functions. Overall, these findings highlight distinct organizational patterns within the cognitive network, influenced by participant handedness.

## Discussion

The primary objective of this study was to uncover interactions between different cognitive functions and domains using network analyses in healthy subjects. Specifically, we aimed to investigate whether modulations in the cognitive network and its interactions could be observed in relation to handedness, a characteristic traditionally associated in specific cases with variability in cerebral network organization despite less distinct differences in cognition and behavioral performance.

Overall, the cognitive network was reconstructed using graph theory, revealing specific cognitive interactions in healthy individuals. Some cognitive functions considered to belong to the same domain exhibited significant correlations with each other. For instance, almost all language functions assessed in this study displayed significant intercorrelations. Importantly, several correlations were also evident between cognitive functions residing in different domains. Among the strongest correlations were those between syntax and switching, as well as between phonology and memory. These results underscore the intrinsic interactive nature of cognition based on mutually supportive functions. Specifically, different processes may account for the strong relationship between syntax and switching. It may be attributed to the sentence-picture matching task's demand for switching between auditory and visual representations, coupled with the need to flexibly navigate across different sentence structures. Previous research has already demonstrated the involvement of executive functions in processing complex or ambiguous syntactic sentences^48–50^. Similarly, the correlation between phonology and memory scores may relate to the necessity to retain the phonological form of two words in memory to perform the rhyme judgment task. Furthermore, the literature has documented the influence of phonological processing abilities on memory performance^51^, supporting the notion of mutual interactions between these functions. These results along with the identified correlations support the concept of cognitive interaction. Our findings showed that language functions are interrelated with executive functions and memory in healthy cognitive functioning, aligning with models emphasizing interactions between language, executive functions, and memory^5,14,52^.

By reconstructing and analyzing the cognitive network with graph theory, we could delineate its organizational characteristics and identify key parameters for understanding this cognitive architecture. Notably, language functions are those that assumed central roles within the network, as evidenced by higher strengths, designating them as hubs in the cognitive network. Indeed, these functions exhibited the most extensive interactions with others, highlighting their central role in healthy cognitive functioning^53^. In contrast, memory, executive functions, and theory of mind, assumed a more peripheral position within the network, tending to operate within clusters. These findings advocate for a more integrative perspective on cognition, revealing the multiple interactions among various functions, dispelling their isolation^14,23^.

The current study also aimed to uncover associations between the cognitive network in healthy subjects and handedness. Interestingly, no significant effects between handedness and cognitive performance (language, memory, executive functions, ToM) were observed. Both right- and left-handed participants exhibited high performance across the administered tasks. This absence of differences aligns with studies asserting that handedness may not significantly be related to behavioral performance^40,41^. Nevertheless, variations in the organization of the cognitive network as a whole were apparent. Correlation patterns exhibited variations between the two groups, highlighting that inter-cognitive interactions varied according to handedness, resulting in distinct cognitive networks. Notably, the cognitive network of left-handed participants exhibited lower values of global metrics in comparisons of right-handed participants. Specifically, the network of left-handed participants demonstrated lower global efficiency, suggesting less prominent overall integration within the cognitive network compared to the right-handed network. Additionally, global clustering coefficient and modularity were also lower for left-handed networks. These findings suggested that the network structure appear to be less segregated and less rigid, with a less localized organization. This trend that manifests at a behavioral level may align with the knowledge that the neurocognitive organization of left-handed individuals presents specific characteristics, including the mobilization of greater interactions between different networks, and for instance increased inter-hemispheric interactions, when performing cognitive tasks^54^. Local analyses further revealed variations in the clustering coefficient, local efficiency, and strength for each function between the two groups. In particular, prosody and syntax presented with the higher strength and assumed the role of the primary hubs in the right-handed network, while semantics played a similarly central role in the left-handed network. Additionally, executive functions, particularly switching and inhibition, appeared more central in the left-handed network. These results are interesting and resonate with other studies that have reported handedness-related associations with certain cognitive processes, reflecting cerebral organization and inter-hemispheric interactions. For example, during grammatical processing in language tasks, left-handedness has been associated with increased reliance on semantic information, whereas right-handedness tends to favor syntactic information^55,56^. Left-handed participants have also shown advantages in executive tasks involving cognitive switching and inhibition^57,58^, although this performance superiority was not consistently observed^59^.

It is worth noting that differences in cognitive network architecture are not necessarily associated with disparities in behavioral or cognitive performance. Instead, these differences may partly explain why such disparities can sometimes be demonstrated. The cognitive network and its interactions may reflect a specific cognitive functioning. One possible hypothesis is that specific cognitive architecture, as unveiled by the network perspective, is likely interdependent with brain architecture and organization, supporting the concept of a cognitome^22,24^. Indeed, in left-handed individuals, cerebral organization has been characterized by reduced asymmetry at anatomical and functional levels^38,39^. Intra- and inter-hemispheric connectivity is increased in left-handers, and morphological distinctions have been highlighted, all indicative of a particular cerebral organization^60–62^. Left-handed individuals, including children, are more likely to exhibit atypical brain organization, particularly within language networks^39,63,64^. The proportion of individuals displaying right-hemispheric specialization for language indeed rises from 4% in strong right-handers to 27% in the most lateralized left-handers^35^. From an integrative perspective, these findings suggest that the neurocognitive organization may differ regarding handedness. Cognitive functioning and interactions vary between left-handed and right-handed individuals, though not in terms of cognitive performance or efficiency. Overall, these findings illustrate an association between handedness and cognitive functioning, which may be interpreted in relation to cerebral organization. In this regard, we hypothesize that the distinct cerebral organization between left-handers and right-handers possibly manifests at two levels. Indeed, at behavioral and motor levels it could be manifested through differences in handedness, and at the cognitive level through variations in profiles and cognitive interactions. In other words, handedness and the cognitive network identified in this study may both be manifestations of a specific cerebral and neurocognitive organization. Further studies are required to investigate the nature and implications of these associations. Overall, this network perspective, using a graph-theoretic approach, has enabled us to identify variations in cognitive functioning that may not be apparent when solely examining behavioral scores. This approach, therefore, offers a complementary lens for gaining a deeper understanding of cognitive processes and the connections forged within neurocognitive networks in general, from a more integrative standpoint.

However, this study has certain limitations. Cognitive interactions were inferred from scores obtained on specific tasks that may not have exclusively assessed the associated functions with absolute purity and specificity. Moreover, only one task was employed to assess each function considered in this study. Future research should explore whether similar findings can be replicated using alternative tasks designed to measure the same functions. This approach would enable to infer that interactions are between functions and not solely attributable to common processes involved in task performance. An important limitation of graph theory applied to cognitive scores concerns the interpretation given to links between nodes. These links are established based on correlation calculations and do not necessarily reflect a concrete association between two cognitive processes. Furthermore, the networks examined in this study are relatively small, consisting of only ten nodes. This limited size makes it challenging to draw robust conclusions based on global metrics extracted. Therefore, any interpretations of the interactions identified in the cognitive network should be made cautiously.

Despite these limitations, this approach offers a broader perspective on the study of cognition. Taken together, the results shed light on the specific interactions among cognitive functions in healthy individuals. The proposed graph-theoretic approach enables a more integrative and precise understanding of cognition. As underscored by the examination of handedness, relying solely on behavioral performance does not fully capture the diverse organization of cognitive interactions. Our findings reveal that, even when performance levels are comparable, the cognitive network exhibits distinct configurations between left-handed and right-handed participants, potentially reflecting differences in cerebral organization. In essence, we have demonstrated that cognition can be explored as a network in which component functions interact in a modular manner^23^. Approaching the study of cognitive organization from this cognitomic perspective holds promise for gaining a deeper understanding of cognition and its underlying interactions in typical individuals, throughout development, and in clinical populations. It is important to view this approach as complementary to others in the field, contributing to a more comprehensive understanding of cognitive and neuro-cognitive functioning.

## Data Availability

The datasets from the study are available from https://osf.io/bx9z3/.
